# Association of preoperative multiparametric MRI with oncological and functional outcomes following robot-assisted radical prostatectomy: a multicentre prospective study

**DOI:** 10.3389/fonc.2026.1795905

**Published:** 2026-05-11

**Authors:** Maria Isabel Galante Romo, Jesús Moreno Sierra, Alejandro Martín Parada, Alberto Palacios Hernández, Pablo Eguiluz Lumbreras, Javier Flores Fraile, Juliusz Jan Szczesniewski, María Fernanda Lorenzo Gómez

**Affiliations:** 1 Urology Department, Clinico San Carlos Hospital, Madrid, Spain; 2 Urology Department, University Hospital of Salamanca, Salamanca, Spain; 3 Surgery Department, University of Salamanca, Salamanca, Spain; 4 Urology Department, University Hospital of Getafe, Getafe, Spain; 5 Department of Medicine, Faculty of Biomedical and Health Sciences, Universidad Europea de Madrid, Madrid, Spain

**Keywords:** mpMRI, prostate cancer, PSA recurrence, surgical planning, tumour staging

## Abstract

**Introduction:**

Multiparametric magnetic resonance imaging (mpMRI) has enhanced the characterisation of prostate cancer (PCa) and its surgical management through robot-assisted radical prostatectomy (RARP). This study analyses the correlation between preoperative mpMRI findings and postoperative clinical outcomes.

**Materials and methods:**

A retrospective analysis of a prospectively maintained multicentre database was conducted, including 1,136 patients who underwent RARP between 2011 and 2022 at two university hospitals. Patients were grouped according to postoperative PSA levels into success (SG) and recurrence (RG) groups. Clinical, imaging, and pathological variables were analysed.

**Results:**

The recurrence rate was 13.6%. RG patients had higher preoperative PSA levels, more T3-stage tumours on mpMRI, more positive surgical margins, and more postoperative complications. Urinary incontinence was significantly more common in RG (49.35% vs. 7.94%). While mpMRI had limited accuracy in predicting tumour extension, it contributed to surgical planning. Multivariable regression identified significant predictors of postoperative success.

**Conclusion:**

mpMRI is useful for patient selection and surgical planning in PCa, though its predictive accuracy still requires optimization.

## 1 Introduction

Prostate cancer (PCa) is one of the most common malignancies among men, and its diagnostic and therapeutic approaches have significantly evolved in recent years. The introduction of multiparametric magnetic resonance imaging (mpMRI) has brought about a substantial shift in the evaluation of these patients, enabling improved disease characterisation and facilitating therapeutic decision-making (1–3). MpMRI has not only enhanced the detection of clinically significant prostate cancer but also allows for the assessment of local disease extent and assists in selecting the most appropriate treatment modality (4–7). Multiparametric MRI combines T2-weighted imaging, diffusion-weighted imaging (DWI), and dynamic contrast-enhanced imaging (DCE) to provide both anatomical and functional information about prostate tissue (8). This improves the detection of clinically significant prostate cancer, enhances lesion localization for targeted biopsies, reduces overdiagnosis of indolent cancers, and supports better staging and surgical planning. Compared to single-sequence MRI, mpMRI offers greater sensitivity and specificity—especially when interpreted by subspecialty-trained radiologists (9).

Among surgical options, robot-assisted radical prostatectomy (RARP) has become the most widely employed technique for patients with localized PCa due to its advantages in precision and functional recovery (10, 11). In cases of locally advanced PCa, the European Association of Urology (EAU) (12) and the National Comprehensive Cancer Network (NCCN) (13) guidelines consider radical prostatectomy as part of a multimodal therapeutic approach in highly selected patients who may benefit from surgery (14). However, evidence regarding the oncological effectiveness of RARP in this context remains limited.

Recent studies have proposed technical modifications to RARP to improve both oncological and functional outcomes in patients with locally advanced PCa. In this regard, Mazzone et al. (15) described a revised version of RARP, referred to as “superextended RARP” (SE-RARP), specifically designed for patients with iT3a or iT3b lesions identified on mpMRI. In their study, the biochemical recurrence rate at two years was 55%, with 66% of patients remaining free from additional treatment during that period (15).

In recent years, RARP has undergone significant technological advancements and technical refinements, improving surgical outcomes (14, 16). Nevertheless, further evaluation is needed regarding the impact of mpMRI on candidate selection for RARP, its influence on surgical planning, and its correlation with oncological and functional postoperative outcomes.

This study contributes to the functional outcomes post-RARP when mpMRI-targeted biopsy is used as a complementary preoperative tool for stratifying patients and guiding surgical planning and ruling out extra-prostatic extension in the surgical treatment.

This study aims to analyse the relationship between preoperative mpMRI findings and the clinical outcomes of robot-assisted radical prostatectomy, with particular emphasis on their impact on cure rate, cancer-specific survival, and the optimization of therapeutic decision-making in prostate cancer management.

## 2 Materials and methods

A retrospective analysis of a prospectively maintained multicentre database was conducted. A total of 1,136 patients diagnosed with prostate cancer and treated with robot-assisted radical prostatectomy (RARP) were included between January 1, 2011, and September 1, 2022, in the University Hospital of Salamanca and the University Clinic Hospital San Carlos in Madrid.

The sample size was calculated using Epidat 4.2 software to compare patients based on outcomes following prostatectomy. A minimum of 130 patients per group was obtained, assuming a heterogeneity of 50%, a margin of error of 5%, and a confidence level of 95%. Participants were subsequently included in a sequential, non-concurrent manner.

Patients were classified in two groups based on postoperative outcomes, defined as PSA persistence (PSA ≥0.1 ng/mL at six weeks after surgery) or biochemical recurrence (PSA≥0.2 ng/mL confirmed on two consecutive measurements):

- Success group (SG): considered as patients without PSA persistence or biochemical recurrence.
- Recurrence group (RG): considered as patients with PSA persistence or biochemical recurrence.

A positive ultrasound was defined as a preoperative transrectal examination that identified suspicious hypoechoic lesions, asymmetry, or irregularities in the prostate capsule, to ensure consistency in technique and interpretation, all ultrasound examinations were performed by board-certified radiologists with experience in prostate imaging, the use of structured reporting and institutional guidelines helped minimise discrepancies. Both participating centres followed standardised imaging protocols, and equipment calibration was routinely maintained.

All MRI studies were interpreted by two radiologists (one from each institution) using 1.5T and 3T equipment, images were systematically interpreted and reported according to the Prostate Imaging Reporting and Data System (PI-RADS) guidelines (versions 2.0 and 2.1, corresponding to the study’s timeline). Surgeries were performed by three experienced surgeons per centre, each beyond the learning curve (each surgeon had a sufficient level of experience to perform the procedure consistently and safely, yielding stable outcomes). All procedures were carried out using the Da Vinci X robotic platform.

Surgical planning was individualised based on a cognitive fusion of mpMRI findings. Surgeons preoperatively reviewed T2-weighted, DWI, and ADC maps to localize index lesions and assess their proximity to the prostatic capsule. This dictated the degree of nerve-sparing (intrafascial vs. interfascial) or the necessity of a wider resection. For patients with suspected extraprostatic extension (iT3a) or seminal vesicle invasion (iT3b) on imaging, a non-nerve-sparing approach or ‘superextended’ RARP was prioritised to ensure negative surgical margins.

Missing data regarding formal MRI T-staging were handled by dichotomizing the variable into ‘MRI Stage T3’ versus ‘Other/Not reported’ to maximize cohort retention in the multivariable model.

Inclusion criteria:

-Male patients over 18 years of age, diagnosed with prostate cancer, who have received primary treatment via prostatectomy.
-Patients with an available, legible, and complete medical record suitable for accurate data collection.
-Patients who have signed informed consent for the use of their clinical data for scientific purposes, and whose treatment and follow-up have been conducted in accordance with Good Clinical Practice Guidelines and current biomedical research legislation.

Exclusion criteria:

-Female patients.
-Patients under 18 years of age.
-Patients with a diagnosis of prostate cancer that has not been confirmed, or who have not received treatment within the established timeframe.
-Incomplete, illegible, or insufficient medical records for proper analysis.
-Patients who have not signed informed consent for the use of their clinical data.

Based on the information contained in the clinical records, the data collection booklet was prepared, along with an electronic Excel document with the studied variables, coded for analysis.

Clinical, demographic, and oncological data were collected: age, body mass index (BMI), time from diagnosis to final follow-up, pre-and postoperative PSA levels (obtained approximately one month prior to the surgical procedure, timing was consistent across the cohort and aligned with standard preoperative evaluation protocols, as for PSA levels after surgery, authors applied same time window), prostate volume, histopathological parameters, surgical complications, postoperative treatments, current patient status, and cancer-specific survival; the dropout rate was less than 5%.

The percentage of tumour volume was determined during routine histopathological evaluation by experienced genitourinary pathologists: total length in millimetres, number of distinct tumour and estimated percentage of tumour involvement in the studied material. The percentage estimation was based on visual assessment of tumour tissue relative to the total prostate volume in the formalin-fixed paraffin-embedded specimen.

Urinary incontinence was evaluated with the International Consultation on Incontinence Questionnaire (ICIQ), and erectile dysfunction was evaluated with the International Index of Erectile Function (IIEF-6), both validated internationally and applicable in multicentre settings.

Functional status was assessed using ICIQ and IIEF-6 questionnaires at baseline and postoperatively (1, 3, 6, and 12 months). Urinary incontinence was defined by ICIQ-validated thresholds.

The results were analysed using statistical tools to ensure meaningful interpretation. Descriptive statistics summarised the data, providing measures of mean, median, standard deviation, and range for continuous variables.

Comparative analyses included the Kruskal–Wallis H test to compare means across multiple groups with non-normally distributed data; and Fisher’s exact test to assess differences in categorical variables.

Multivariable logistic regression was conducted to identify independent predictors of postoperative outcomes, specifically surgical success and PSA persistence, while controlling for potential confounding factors. The effect size for these associations was estimated and is reported as Odds Ratios (OR) alongside their corresponding 95% confidence intervals (CI). For categorical predictors, the absence of the clinical finding (e.g., negative ultrasound) or the lowest clinical/pathological stage (e.g., PI-RADS 0-2) was consistently defined as the reference category to ensure standardised clinical interpretability. Missing data was strictly handled using listwise deletion (complete case analysis) within the regression models to prevent imputation bias.

Logistic regression was restricted to fixed-timepoint outcomes (PSA persistence at six weeks). To adjust for variable follow-up and censoring in long-term biochemical recurrence, time-to-event analyses (Kaplan-Meier and Cox regression) were employed; to prevent overfitting and multicollinearity, the models maintained an Events Per Variable (EPV) ratio > 15 and a Variance Inflation Factor (VIF) < 1.5 for all predictors.

Statistical analysis was performed using IBM Corp. Released 2017. IBM SPSS Statistics for Windows, Version 25.0. Armonk, NY: IBM Corp. Statistical significance was set at p < 0.05.

While the data extraction and cohort assembly were performed retrospectively, patient evaluation, RARP procedures, and scheduled postoperative follow-ups were conducted according to prospective, standardised institutional protocols at each participating centre between 2011 and 2022. This design aimed to reflect the evolution of clinical practice, including the progressive adoption of technologies such as multiparametric MRI and novel surgical techniques. To minimise potential biases, stratified analyses by centre and inclusion period were performed, and the consistency of outcomes was assessed through sensitivity analyses. The authors acknowledge the inherent limitations associated with technological and clinical advancements, which were carefully considered and adjusted for in the analysis, thereby enhancing the generalisability of the findings across diverse clinical settings.

In the presence of sparse data and extreme estimates, a sensitivity analysis using penalized logistic regression was performed to assess the stability of the model.

The study was approved by the Ethics Committee for Research with Medicinal Products (CEIM, Ref. 2022/09). Patient confidentiality was protected in accordance with applicable regulations (Spanish Biomedical Research Law 14/2007 and EU Directive 2001/20/EC). All patients provided informed consent authorizing the use of their clinical data for research purposes. The study was conducted without financial incentives and without altering standard clinical practice.

This study was conducted within the framework of the GRUMUR research group; however, no specific funding, financial support, or external funding was received.

## 3 Results

### 3.1 General patient characteristics

A total of 1,136 patients undergoing robot-assisted radical prostatectomy were included.

The mean age was 63.37 years (SD ± 6.27, range 41-78, df 1), with no significant differences between SG (mean 63.42) and RG (mean 63.06) p = 0.514. The mean body mass index (BMI) was 27.40 kg/m² (SD ± 3.58, range 19.03-41.98, df 1), SG (mean 27.37) and RG (mean 27.54), with no statistically significant differences (p = 0.948), Kruskal–Wallis H test.

### 3.2 Follow-up and preoperative parameters

The mean follow-up time was 1,117.51 days (SD ± 608.57, range 90.00-4,081.00, df 1), significantly longer in the RG mean 1,402.90 (SD ± 692.46, range 117.00-3809.00, df 1) compared to the SG mean 1,080.96 (SD ± 587.57, range 90.00-4,081.00, df 1); p <0.0001).The mean interval between biopsy and treatment was 5.87 months (SD ± 9.36, range 0.13-102.20, df 1), SG mean 5.93 months (SD ± 9.51, range 0.13-102.20, df 1) in comparison to RG mean 5.47 months (SD ± 8.16, range 0.89-65.24, df 1), no significant difference was found between groups (p = 0.946).

The mean interval between mpMRI and treatment was 2.60 months (SD ± 0.86, range 0.13-3.98, df 1), SG mean 2.57 months (SD ± 0.87, range 0.13-3.98, df 1) in comparison to RG mean 2.74 months (SD ± 0.74, range 0.89-3.91, df 1), no significant difference was found between groups (p = 0.215).

The Charlson comorbidity index had a mean of 2.42 (SD ± 1.00, range 0.00-9.00, df 1), SG mean 2.41 (SD ± 1.00, range 0.00-9.00, df 1) in comparison to RG mean 2.50 (SD ± 0.96, range 0.00-5.00, df 1), also without relevant differences (p = 0.186).

The mean pre-treatment PSA was 7.83 ng/ml (SD ± 6.07, range 0.23-62.91, df 1), it was significantly higher in RG mean 9.26 (SD ± 9.62, range 0.23-62.91, df 1) in comparison to SG mean 7.61 (SD ± 5.29, range 0.23-55.26, df 1), with significant differences (p=0.007).

The number of biopsy cores positive for malignancy was similar between groups, However, the total number of biopsy cores obtained was higher in the SG mean 3.82 (SD ± 2.76, range 1.00-16.00, df 1) in comparison to RG mean 4.04 (SD ± 2.72, range 1.00-13.00, df 1), p = 0.281, Kruskal–Wallis H test.

### 3.3 Surgical and postoperative findings

Total hospital stay was 5.09 days (SD ± 2.94, range 1.00-30.00, df 1), RG patients (mean 5.73, SD ± 3.94, range 2.00-30.00, df 1) had a longer postoperative hospital stay than SG patients mean 4.97 (SD ± 2.71, range 1.00-30.00, df 1) p = 0.032.

The main prostate weight was 47.10 grams (SD ± 18.51, range 1.00-118.00, df 1) and no differences across groups SG mean 47.10 grams (SD ± 18.54, range 1.00-115.00, df 1), RG mean 47.11 grams (SD ± 18.43, range 19.50-118.00, df 1); p = 0.954.

The mean post-treatment PSA was 0.24 ng/ml (SD ± 1.21, range 0.01-14.49, df 1), it was higher in RG mean 1.20 (SD ± 2.54, range 0.01-12.63, df 1) in comparison to SG mean 0.11 (SD ± 2.54, range 0.01-14.49, df 1), with significant differences (p=0.006).

Interestingly, the percentage of tumour volume in the surgical specimen was higher in the SG mean 32.10 (SD ± 21.83, range 0.90-95.00, df 1) than in the RG mean 19.83 (SD ± 19.42, range 2.00-85.00, df 1); p= 0.0006, Kruskal–Wallis H test.

### 3.4 Imaging and pathological findings

The SG showed a higher proportion of early-stage tumours on mpMRI, particularly T1 (6.31% vs. 1.95%; p = 0.0251), T2b (13.03% vs. 5.84%; p = 0.0108), and T2c (15.27% vs. 5.84%; p = 0.0010), whereas the RG showed more stage T3 tumours (18.18% vs. 9.67%; p = 0.0031).

Pathological staging also differed significantly: the SG had more stage T2a (20.26% vs. 2.6%; p = 0.0001) and T3a (20.06% vs. 9.09%; p = 0.0008), while the RG had more T2b tumours (43.51% vs. 13.34%; p = 0.0001). No significant differences were found for pathological stage T2c (p = 0.6526) or T0 (p = 0.1385).

The RG had a significantly higher rate of positive surgical margins (72.73% vs. 38.8%; p = 0.0001, **Table 1**), Fisher’s exact test.

**Table 1:** Imaging and pathological findings.

Groups	Success group n=982		Recurrence group n=154		Total, n=1136		P-value
MRI stage	n	%	n	%	n	%	
T1	62	6.31	3	1.95	65	5.72	0.0251
T2a	147	14.97	14	9.09	161	14.17	0.0614
T2b	128	13.03	9	5.84	137	12.06	0.0108
T2c	150	15.27	9	5.84	159	14.00	0.0010
T3	95	9.67	28	18.18	123	10.83	0.0031
Missing values	400	40.73	91	59.09	491	43.22	NA
Pathological Stage T		%		%			
T2a	199	20.26	4	2.60	203	17.87	0.0001
T2b	131	13.34	67	43.51	198	17.43	0.0001
T2c	355	36.15	59	38.31	414	36.44	0.6526
T3a	197	20.06	14	9.09	211	18.57	0.0008
T3b	76	7.74	4	2.60	80	7.04	0.0171
T0	3	0.31	2	1.30	5	0.44	0.1385
Missing values	21	2.14	4	2.60	25	2.20	NA

### 3.5 Gleason score patterns

Significant differences were observed between SG and RG in biopsy Gleason scores. While proportions of Gleason 3 + 3 (39.82% vs. 39.61%; p = 1.0000), 4 + 3 (9.78% vs. 9.74%; p = 1.0000), and 5 + 4 (3.36% vs. 3.25%; p = 1.0000) were similar, Gleason 4 + 4 was more frequent in the RG (14.29% vs. 5.4%; p = 0.0002). There was a trend toward higher frequencies of 4 + 5 (3.9% vs. 1.53%; p = 0.0534) and 3 + 5 (2.6% vs. 0.92%; p = 0.0868) in the RG. Interestingly, Gleason 5 + 5 was more frequent in the SG (5.7% vs. 0.65%; p = 0.0044), possibly due to classification bias or low case numbers (**Table 2**), Fisher’s exact test.

**Table 2:** Gleason score patterns.

Groups	Success group n=982		Recurrence group n=154		Total, n=1136		p-value
Biopsy Gleason	n	%	N	%	n	%	
3 + 3	391	39.82	61	39.61	452	39.79	1.0000
3 + 4	181	18.43	22	14.29	203	17.87	0.2575
4 + 3	96	9.78	15	9.74	111	9.77	1.0000
4 + 4	53	5.40	22	14.29	75	6.60	0.0002
4 + 5	15	1.53	6	3.90	21	1.85	0.0534
5 + 4	33	3.36	5	3.25	38	3.35	1.0000
5 + 5	56	5.70	1	0.65	57	5.02	0.0044
3 + 5	9	0.92	4	2.60	13	1.14	0.0868
Missing values	148	15.07	18	11.69	166	14.61	

### 3.6 Complications and adverse outcomes

The RG experienced a higher rate of surgical complications compared to the SG (13.64% vs. 2.34%; p = 0.0001), including bladder injury (5.19% vs. 0.81%; p = 0.0005), intraoperative haemorrhage (3.25% vs. 0.71%; p = 0.0153), and bowel injury (3.25% vs. 0.31%; p = 0.0017).

Urinary incontinence was significantly more common in the RG, with 49.35% requiring specialized consultation versus 7.94% in the SG (p = 0.0001), Fisher’s exact test.

The mean time for urinary incontinence and erectile dysfunction was 1.30 months (SD ± 0.10, range 0.10-1.50, df 1), no significant difference was found between groups (p = 0.521).

### 3.7 Current status and survival

At the end of the follow-up period, 98.68% of patients were alive, with no significant differences between groups (p = 1.0000).

Multivariable regression (**Table 3**) identified several factors significantly associated with surgical success; there was no collinearity. Predictors of favourable outcomes included absence of prior prostate treatment (p = 0.035), PIRADS 0–2 on MRI (p = 0.038), and pathological stages T2a (p = 0.005) and T3a (p = 0.0001). In contrast, positive preoperative ultrasound (p = 0.0001), clinical stage T2c (p = 0.0001) or T3 (p = 0.0002), and conversion to open/laparoscopic surgery (p = 0.005) were associated with poorer outcomes. **Table 3**.

**Table 3:** Multivariable regression.

Variables	Total, n=1136		Odds Ratio (OR)	P-value	95% I.C lower	95% I.C upper	Collinearity VIF
	n	%					
Absence of previous prostate treatment	1014	89.26	0.09	0.035	0.01	5.986	1.017
Positive ultrasound	206	18.13	4.0	0.0001	0.44	4.746	1.138
Clinical stageT2c	29	2.55	2.08	0.0001	1.00	4.322	1.024
Clinical stageT3	72	6.34	6.46	0.0002	0.34	9.765	1.368
PIRADS 0 - 2	155	13.64	0.15	0.04	0.02	0.99	1.018
Conversion to open or laparoscopic surgery	136	11.97	1.86	0.005	0.16	2.800	1.515
Pathological Stage T2a	203	17.87	0.64	0.005	0.19	2.189	1.116
Pathological Stage T3a	211	18.57	0.25	0.0001	0.001	3.328	1.088

Some odds ratio estimates in **Table 3** present wide confidence intervals, which reflect variability in subgroup sizes and the distribution of events. The odds ratio and corresponding 95% confidence interval for PI-RADS 0–2 have been corrected to ensure internal consistency.

### 3.8 PSA persistence

Multivariable regression identified several factors significantly associated with PSA persistence; Omnibus test p < 0.0001, chi-square 628.86, Nagelkerke R² 0.776, with no evidence of collinearity (df = 9), and 77.60% of subjects correctly classified by the model.

**Figure 1:**
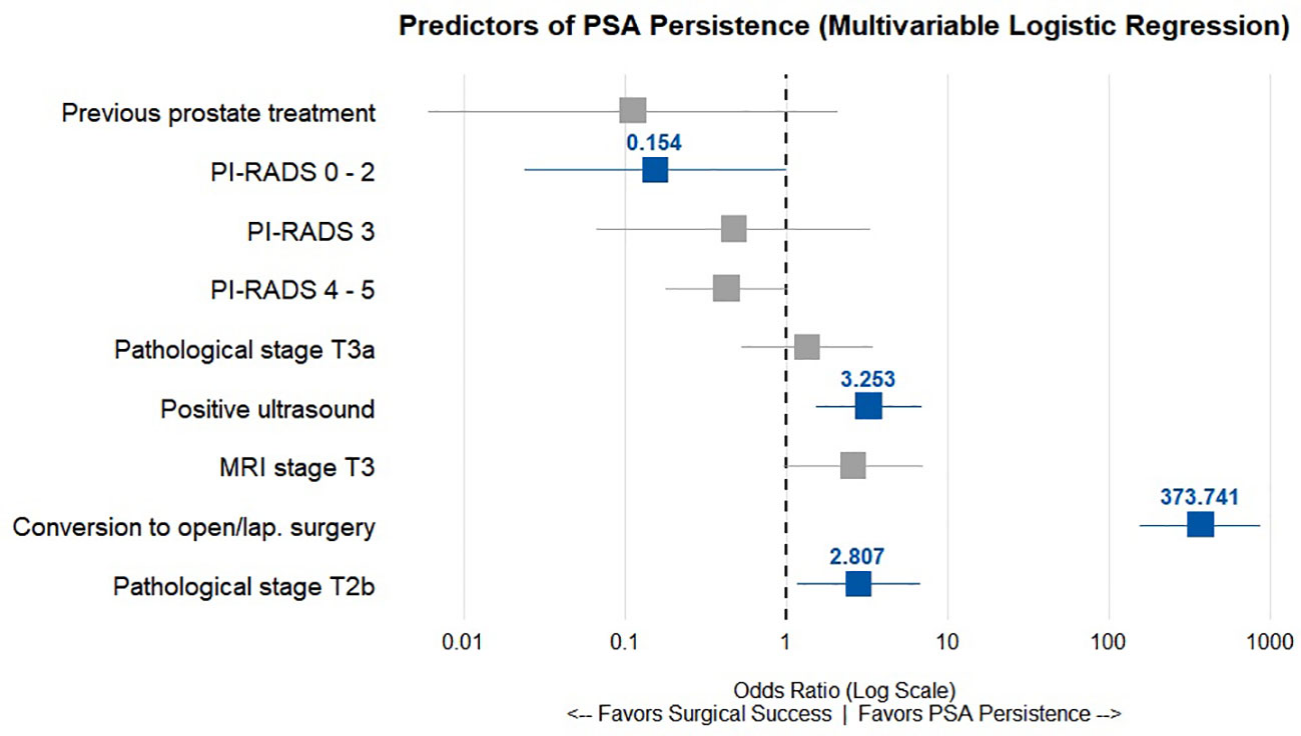
Odds Ratio (OR) of factors correlated with persistent PSA levels.

The main independent predictors associated with higher PSA persistence included the presence of a positive preoperative ultrasound (OR 3.25, p = 0.002), pathological tumour stage T2b (OR 2.81, p = 0.021), and conversion to open or laparoscopic surgery (OR 373.74, p < 0.001). In contrast, a preoperative MRI finding of PI-RADS 0–2 was the only variable significantly associated with lower PSA persistence (OR 0.15, p = 0.049) (**Table 4**; **Figure 1**).

**Table 4:** Odds Ratio (OR) of factors correlated with persistent PSA levels.

Variables	Beta coefficient	Standard error	Wald	P-value	Odds Ratio (OR)	95% C.I. lower	95% C.I. upper	Collinearity VIF
Previous prostate treatment	-2.191	1.492	2.156	0.142	0.112	0.006	2.082	1.075
PIRADS 0 - 2	-1.868	0.949	3.872	0.049	0.154	0.024	0.993	1.164
PIRADS 3	-0.747	0.997	0.562	0.454	0.474	0.067	3.343	1.061
PIRADS 4-5	-0.853	0.447	3.637	0.057	0.426	0.177	1.024	1.174
Pathological Stage T3a	0.295	0.479	0.381	0.537	1.344	0.526	3.435	1.061
Positive ultrasound	1.180	0.386	9.358	0.002	3.253	1.528	6.926	1.166
MRI stageT3	0.956	0.512	3.490	0.062	2.601	0.954	7.087	1.066
Conversion to open/laparoscopic surgery	5.924	0.441	180.629	< 0.001	373.741	157.546	886.615	1.272
Pathological Stage T2b	1.032	0.448	5.303	0.021	2.807	1.166	6.756	1.156

Given the substantial missingness of formal MRI staging, a sensitivity analysis excluding this variable was performed. The independent predictive value of the remaining clinical and imaging parameters—particularly PI-RADS classification and positive ultrasound—remained statistically robust, supporting the stability of the model.

The extremely large odds ratio observed for conversion to open/laparoscopic surgery is likely related to sparse data and potential quasi-complete separation and should therefore be interpreted with caution.

A sensitivity analysis using penalized regression yielded consistent directional results, supporting the robustness of this association.

### 3.9 Biochemical recurrence

**Figure 2:**
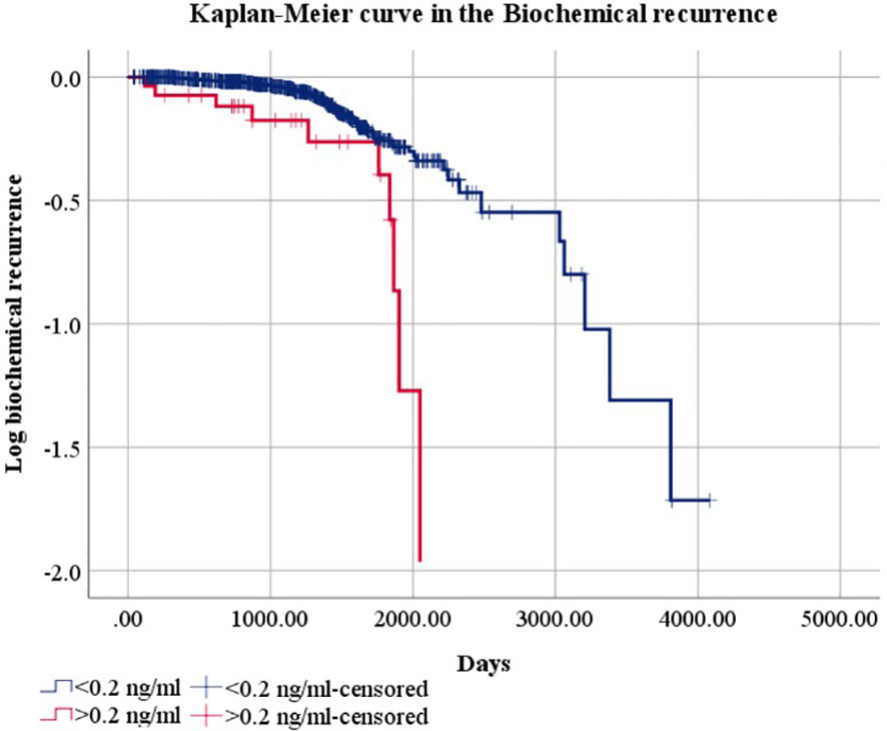
Kaplan–Meier curve.

In the Kaplan–Meier analysis, a significant difference in biochemical recurrence-free survival was observed between groups (Mantel–Cox test: 15.394, df = 1, p = 0.0001), as shown in **Figure 2**. Patients with PSA levels > 0.2 ng/mL exhibited a faster progression compared to those with PSA < 0.2 ng/mL.

Median survival estimates and corresponding confidence intervals were carefully re-evaluated to ensure internal consistency, and all reported values have been corrected accordingly in **Figure 2**.

Cox regression analysis revealed that biochemical recurrence was associated with a higher prevalence of clinical stage T2c (B = 3.891, p = 0.0001, Exp(B) = 48.93, 95% CI: 6.63–361.08), clinical stage T3 (B = 3.356, p = 0.0001, Exp(B) = 28.67, 95% CI: 10.58–77.71), and a lower prevalence of stage T2b as determined by magnetic resonance imaging (B = -2.223, p = 0.001, Exp(B) = 0.10, 95% CI: 0.029–0.407).

## 4 Discussion

This multicentre study based on a retrospective analysis of a prospectively maintained database evaluates the relationship between mpMRI findings and oncological and functional outcomes following RARP, the persistence of positive surgical margins (PSM), biochemical recurrence, and urinary incontinence remain significant challenges.

In our cohort of 1,136 patients undergoing RARP, recurrence occurred in 13.6% and was significantly associated with higher preoperative PSA, elevated Gleason scores, and a higher rate of positive margins. In the recurrence group, mpMRI findings were associated with a higher prevalence of T3 stage tumours (18.18% vs. 9.67%; p = 0.0031) and positive surgical margins (72.7% vs. 38.8%; p = 0.0001). These results underscore the limitations of mpMRI when used as a standalone tool for staging and surgical planning. This highlights the need for a multimodal approach that integrates imaging findings with clinical and pathological data to optimize preoperative assessment and surgical strategy.

Although the observed difference in PSA levels between groups (7.61 vs. 9.26 ng/mL) reached statistical significance, its clinical relevance remains uncertain. PSA is a widely used biomarker in prostate cancer assessment, that may imply differences in tumour biology, burden, or aggressiveness, however, the absolute difference in this case is modest and may not, by itself, influence clinical decision-making or alter treatment strategies, future studies with larger cohorts and long-term follow-up are needed to determine whether such PSA differences are associated with oncologic outcomes or perioperative parameters.

While mpMRI is central, other factors as PSA levels, Gleason score, timelines, also play a role in predicting extraprostatic extension, tumour aggressiveness, and surgical risk, mpMRI improves this assessment by providing visual confirmation of lesion location, size, and potential extraprostatic extension, which is especially valuable for decisions during surgery, similar findings were concluded in a study by Mohammad Hossein Jamshidi et al, where standardised protocols and checklists for mpMRI interpretation are essential for achieving greater consensus, leading to improved diagnostic outcomes in prostate cancer (17).

While radiological T3 findings are intrinsically linked to aggressive tumour biology, our multivariable analysis confirmed that mpMRI findings—particularly the PI-RADS classification—act as independent predictors of surgical success and PSA persistence, providing critical information for preoperative risk stratification beyond conventional clinical parameters.

Preoperative mpMRI influences functional outcomes indirectly by guiding surgical strategies. Thus, image-guided surgical adjustments—rather than imaging itself—are the direct determinants of postoperative functional recovery.

Several studies have assessed the diagnostic accuracy of mpMRI in local staging of PCa. A meta-analysis by de Rooij et al. (18), reported high specificity (91–96%) for detecting extracapsular extension (ECE), seminal vesicle invasion (SVI), and stage T3 disease, but with limited and heterogeneous sensitivity (57–61%). Although the addition of functional techniques (DWI, DCE) and use of 3T magnets modestly improved sensitivity, endorectal coils offered no clear advantage. Chandrasekar et al. (19) analysed patients in a regional registry and found even lower sensitivities (16.8–37%) for detecting ECE, SVI, and nodal involvement, though specificity remained above 90%. In both studies, sensitivity was higher in high-risk patients suggesting the clinical utility of mpMRI is highly context dependent.

Our findings are consistent with these reports. The ability of mpMRI to correctly predict ECE in our cohort was limited; although 18.18% of patients in the RG were staged as T3, this was significantly higher than in the SG (9.67%),the RG showed a markedly high rate of positive margins.

To improve preoperative prediction of ECE, predictive models have been developed that integrate imaging, clinical, and biopsy data. A recent study involving MRI-targeted biopsies prostate lobes proposed a lateralized nomogram including PSA, index lesion diameter, ISUP grade, and percentage of positive ipsilateral cores. In this model, the presence of ECE on MRI had an odds ratio of 3.74 for confirmed ECE in surgical specimens (20). Our study aligns with some of these factors, showing a significant higher proportion of Gleason 4 + 4 in the recurrence group.

Our cohort showed a significantly higher rate of urinary incontinence in the RG (49.35% vs. 7.94%; p = 0.0001). In this context, membranous urethral length (MUL) as measured by MRI has been identified as a key predictor of functional recovery. Kim et al. demonstrated that each additional millimetre in MUL significantly increases the likelihood of postoperative continence, reinforcing the added value of MRI in functional planning (21).

Finally, advanced technologies such as 3D surgical planning models have shown promise in improving both oncological and functional outcomes. Shirk et al. (22) reported a significant reduction in biochemical recurrence and need for adjuvant or salvage therapy in patients whose surgeries were guided by 3D models, pointing toward a promising future in precision surgery.

Gleason score was determined based on pre-operative biopsy results to evaluate and compare baseline tumour characteristics available at the time of diagnosis and prior to definitive treatment. Biopsy Gleason score remains a key factor in clinical decision, risk stratification, and surgical planning, it was essential to include this variable in our analysis to reflect real-world preoperative assessment. Using biopsy data allowed us to assess potential differences in diagnostic presentation between groups, which may influence treatment timing and approach.

Regarding the variability in biopsy-to-surgery timing, the observed differences in Gleason patterns, may reflect true biological differences or sampling variability rather than temporal progression. We have added a paragraph in the discussion to acknowledge this limitation and suggest that future studies.

Future research should explore the incorporation of artificial intelligence and biomarkers into preoperative decision-making processes to further optimize outcomes in patients undergoing surgical treatment for prostate cancer.

This study provides one of the most comprehensive multicentre evaluations to date of mpMRI’s role in robotic-assisted radical prostatectomy (RARP), drawing on a large cohort of 1,136 patients over an 11-year period. Unlike previous studies with limited scope or short-term follow-up, our analysis reflects real-world clinical practice and incorporates longitudinal outcomes. The novel stratification of patients based on postoperative PSA levels allowed for a clinically meaningful assessment of mpMRI’s predictive value. Through multivariable modelling, we identified independent predictors of surgical success and PSA persistence, integrating imaging, pathological, and clinical variables. While mpMRI proved valuable in guiding surgical planning, particularly in risk stratification and margin control, its limited accuracy in predicting tumour extension underscores the need for protocol optimisation and the potential integration of AI-based tools to enhance diagnostic precision.

This study has several limitations to consider. Although this study is based on a retrospective analysis of a prospectively maintained multicentre database, the inclusion of patients from different institutions may have introduced variability in surgical technique and mpMRI interpretation. Despite standardised protocols, subjectivity in mpMRI evaluation and tumour staging may have affected margin and recurrence predictions. Additionally, the lack of a uniform postoperative functional assessment protocol—particularly for continence—may have led to differences in data collection between centres. Lastly, while this study supports the utility of mpMRI for preoperative staging, the integration of artificial intelligence and biomarkers into predictive models remains unexplored and represents a promising avenue for optimizing surgical decision-making.

## 5 Limitations

The 12-year study period (2011–2022) poses a potential temporal bias. However, the use of standardised PI-RADS reporting by expert uroradiologists and the high surgical volume of the participating centres ensured consistent diagnostic and technical quality throughout the cohort.

Furthermore, potential centre-related bias was minimized by standardised protocols. A sensitivity analysis incorporating the surgical institution into the multivariable model confirmed that the hospital centre was not a significant predictor of oncological outcomes (p = 0.784).

Additionally, some regression estimates—particularly for conversion to open/laparoscopic surgery—showed extreme values due to sparse data and possible quasi-complete separation. Although sensitivity analyses using penalized regression confirmed the direction of the association, these results should be interpreted cautiously.

## 6 Conclusion

The findings of this study confirm the relevance of multiparametric magnetic resonance imaging (mpMRI) in the preoperative assessment of prostate cancer and its role in guiding the diagnostic process and surgical planning for robot-assisted radical prostatectomy (RARP).

MpMRI facilitated more accurate risk stratification; however, the high rate of positive surgical margins (72.7% in the recurrence group) highlights the need to improve its predictive accuracy.

Overall, these findings highlight the importance of integrating clinical, radiological, and pathological parameters into surgical planning, and demonstrate how differences in tumour profile can influence both functional and oncological outcomes following robotic prostatectomy.

Despite the recognized oncological and functional benefits of RARP, our results underscore the importance of integrating advanced predictive models and personalized surgical strategies to reduce biochemical recurrence and improve cancer-specific survival.

## Data Availability

The raw data supporting the conclusions of this article will be made available by the authors, without undue reservation.
